# A Prospective, Single‐Center Trial Evaluating the Effectiveness of ClimateCare in the Acute Postoperative Management of Pressure Ulcers

**DOI:** 10.1002/hsr2.70846

**Published:** 2025-06-23

**Authors:** Stuti P. Garg, Anitesh Bajaj, Krish V. Shah, Emmanuelle Hanna, Geneviève L. Putnam, Iris Bai, Diana Griffin, Robert D. Galiano

**Affiliations:** ^1^ Division of Plastic & Reconstructive Surgery Northwestern University Feinberg School of Medicine Chicago Illinois USA; ^2^ Case Western Reserve University School of Medicine Cleveland Ohio USA

**Keywords:** BMI, ClimateCare, complications, pressure ulcer, surgical closure

## Abstract

**Background and Aims:**

Pressure ulcers (PU) are injuries to the skin and underlying tissue that can have significant morbidity with the presence of complications such as dehiscence and necrosis. ClimateCare is a mattress coverlet system that aims to maintain optimal skin moisture, temperature, and humidity levels at the interface between the patient and the surface to mitigate pressure ulcer risk factors. The objective of this study is to evaluate the effectiveness of ClimateCare in improving wound outcomes and minimizing complications of pressure ulcers.

**Methods:**

Patients with a stage III/IV pressure ulcer admitted for surgical closure were included in the randomized‐controlled trial. All patients received the Fluid Immersion Simulation (FIS) System, either with or without the ClimateCare treatment based on a convenience sampling method. The subjects were monitored for 14 days post‐closure (POD‐14) for assessment of wound status and complications, including moisture, maceration, drainage, dehiscence, epidermolysis, necrosis, and demarcation.

**Results:**

A total of 32 patients completed the study, where 18 patients received the ClimateCare treatment and 14 patients did not. In the control group, 71% of patients had complications while 17% had complications in the ClimateCare group (*p* = 0.001). In addition, 33% of patients without the ClimateCare had open wounds, while no patients who received ClimateCare treatment had open wounds (*p* = 0.01). Patient acceptability regarding treatment comfort, difficulty with mobilization, and pain at surgical site were not significantly different between ClimateCare and control groups.

**Conclusion:**

Our findings suggest that the ClimateCare treatment in conjunction with the FIS may be effective in decreasing risk of postoperative complications and emphasize the importance of moisture control and pressure offloading in patients. Future studies should be conducted to characterize the effects of ClimateCare in minimizing the risk of complications following wound closure.

## Summary

- Pressure ulcers can have significant morbidity with the presence of complications such as dehiscence and necrosis.
- The objective of this study is to evaluate the effectiveness of the ClimateCare mattress coverlet system in minimizing complications of pressure ulcers, which aims to maintain optimal skin moisture, temperature, and humidity.
- Complications in the ClimateCare group were significantly decreased at 17% compared with the control group at 71%.
- Wound status was also significantly better at 0% open wounds in the ClimateCare group, compared to 33% in the control.

## 1 Introduction

Prolonged pressure to bony prominences of the skin decreases perfusion resulting in ischemia and eventual necrosis of the surrounding tissue [1, 2]. These injuries, known as pressure ulcers (PUs), affect more than 2.5 million people in the United States each year [3]. PUs disproportionately impact adults with limited mobility, malnutrition, incontinence, and decreased sensation [2, 4, 5]. The National Pressure Ulcer Advisory Panel (NPUAP) is a commonly used staging system for PUs based on severity [2]. Open wounds (Stage III or IV) have increased susceptibility to infection and require urgent medical intervention, potentially necessitating surgical flap wound closure [2, 6].

Effective treatment and prevention strategies are essential for reducing the progression of PUs and improving patient outcomes, including the use of pressure‐relieving devices and regular patient repositioning [2, 7]. A fluid immersion simulation system (FIS) is a pressure offloading surface that uses 3D immersion technology to simulate a fluid environment while maintaining near‐homeostatic blood flow and tissue oxygenation levels [6, 8]. In contrast to more standard devices such as an air‐fluidised bed (AFB) which require manual patient repositioning by healthcare staff, a FIS is a fully autonomous system that constantly readjusts based on patient movement [6, 8].

The skin microclimate, including moisture, temperature, and airflow next to the skin surface, is an indirect risk factor in the progression of skin ulcers [9]. These factors can modulate the damage thresholds for the skin and the potential for soft tissue deformation [9, 10]. High moisture levels may cause the wound to become macerated, increase infection susceptibility, and delay healing [1, 2, 9, 10, 11]. Furthermore, wounds exposed to high temperatures may precipitate ischemia, leading to necrotic tissue formation [9, 10]. While a promising technology, FIS lacks microclimate regulation when used alone, which may hinder its therapeutic utility for PU's [6, 8]. With the aim of addressing this issue, a microclimate regulation device such as ClimateCare can be used in conjunction with many support systems including FIS. ClimateCare is a mattress coverlet system that helps maintain optimal skin moisture, temperature, and humidity levels at the interface between the patient and the surface [12]. Through moisture vapor transfer and temperature regulation, it is designed to improve clinical outcomes while enhancing patient comfort. However, while the effect of moisture management in healing after PU closure is well‐accepted, more evidence is needed to establish the utility of moisture control in decreasing healing complications during PU closure.

Another benefit of ClimateCare is its user‐friendly implementation for nurses and care teams. ClimateCare is a single patient use coverlet with minimal assembly needed. While current prevention strategies such as AFB require frequent manual patient repositioning, establishing the clinical utility of ClimateCare with the fully autonomous FIS offers a potential means of PU prevention that is more effective and less time‐consuming. Nurse and patient‐reported satisfaction with treatment modalities are indicators of care quality [12, 13]. Therefore, these metrics should be considered when evaluating a treatment option.

The ability of ClimateCare to regulate microclimate risk factors makes it a promising therapeutic tool, with the potential to increase the clinical utility of FIS for the treatment and prevention of PUs. Our team has previously published preliminary data on the effectiveness of ClimateCare in the acute postoperative management of PUs [14]. However, the goal of this study is to evaluate the long‐term impact of ClimateCare in preventing complications. Through a prospective, controlled trial assessing wound outcomes with and without ClimateCare, we aim to elucidate the therapeutic potential for ClimateCare in the treatment of pressure ulcers.

## 2 Methods

Our study is a prospective, single‐center, human subject trial comparing the use of FIS with and without ClimateCare treatment. Subjects with pressure ulcers were screened for inclusion and exclusion criteria and assigned using convenience sampling to a treatment group immediately after surgical wound closure. Assigned treatments were given and followed for 1 year thereafter, and outcomes between the two groups were compared. This study was reviewed and approved by Northwestern University's Institutional Review Board (STU00200584). All protocols for the use of human subjects were observed in accordance with the IRB. The General Data Protection Regulation (GDPR) and human subject research rules were followed in accordance with Northwestern's IRB.

### 2.1 Subject Recruitment and Criteria

Patients were recruited from Northwestern Memorial Hospital through clinic admission, direct transfer from another facility, or the emergency room. Eligible participants were between 18 and 85 years old and met the study's inclusion and exclusion criteria.

To be included in the study, patients needed to have a Stage III or IV pressure ulcer (PU) that met established clinical criteria. Additionally, they could not have participated in any other clinical trial within 30 days before consent. If the wound had been previously treated, participants were required to have a 30‐day wound history available. Finally, the patients had to be deemed by the investigators to be reasonably compliant with study protocols. Once determined to be eligible, patients met with the Principal Investigator (PI) to discuss the study and signed an Informed Consent Form.

Patients were excluded from the study if they had an estimated life expectancy of less than 12 months, were medically unfit for surgery for any reason, or had a history of radiation therapy. Further exclusions included individuals who, in the opinion of the PI, were noncompliant with medical care or had undergone more than three previous closures of PUs at the same site. Additionally, individuals with a history of bleeding disorders or those experiencing severe fecal incontinence were excluded.

Convenience sampling was used for this study, as this protocol was initially a portion of a separate study, where the ClimateCare surface was added to FIS treatment partway through the study to optimize treatment and care [14]. The treatment assignment for whether the patient would receive FIS therapy alone or FIS with ClimateCare was blinded before study initiation, and no patients were informed of their treatment assignment. A total of 38 subjects were enrolled in the study. Of these, the first 18 patients were assigned to the control group and received FIS therapy alone, while the remaining 20 patients received FIS therapy with ClimateCare.

### 2.2 Product Specifications and Manufacturer Details

The ClimateCare system is a microclimate management mattress coverlet designed to regulate temperature and moisture at the patient‐support surface interface. It functions by removing excess heat and moisture, with a moisture vapor transfer rate of 130 g/m²/hr. The system is compatible with various therapeutic support surfaces, including the Dolphin Fluid Immersion Simulation (FIS) System and the P.R.O. Matt Plus. ClimateCare, is available in multiple sizes (36″, 42″, and 48″ widths), and includes both reusable and disposable components to support infection control protocols. The system is manufactured by Joerns Healthcare, a company based in Charlotte, North Carolina, USA specializing in medical equipment and patient care.

### 2.3 Study Procedures

For each subject, the PI performed wound assessment for appropriateness regarding definitive closure. Only one wound per subject was included in the study. Subjects with multiple wounds were assessed by the PI, who selected the most appropriate wound to include in the study. Immediately after wound closure, subjects received assigned study therapy (FIS with or without ClimateCare) and remained hospitalized or were transferred to a step‐down facility for at least 14 days following their surgical procedure according to their requirements.

During the subject's hospital admission, interventions other than the support surface utilized were based on the institutional standard of care practices. After the initial wound closure, the PI determined if additional surgical debridement was required based on ulcer appearance and periodic post‐debridement culture results. If a flap failed during this period, the subject was removed from the study and transitioned to standard care. For subjects with multiple PUs, any PUs not selected for the study were not followed, but still received standard wound care by the treating clinician. The standard postoperative care included wound care protocols, management of pain, laboratory monitoring, and tailored antibiotics in accordance with culture results. There was also appropriate continuation of home medications, which varied per patient. After the inpatient stay, subjects were followed monthly for 1 year (±20 days) to evaluate the incidence of complications and need for additional therapeutic interventions.

### 2.4 Data Collection

Patient data were extracted from electronic medical records, at bedside during subjects' hospitalization, or through external facilities' staff. A focused medical and surgical history, physical exam, wound history, including the onset and chronicity of the wound and anatomic location, as well as prior wound‐related surgeries and treatments were obtained and recorded. Wounds were measured before debridement following NPUAP recommendations by length, width, and depth using a ruler. After the debridement, the wound was irrigated with 5 L of normal saline and measured again. Digital photographs of the wound were taken at the pre‐debridement stage and following initial debridement. The primary outcome was the complication rate between the two treatment groups (FIS alone vs. FIS with ClimateCare). During the initial 14‐day follow‐up period, data on complications such as moisture, maceration, drainage, dehiscence, epidermolysis, necrosis, and demarcation were recorded. Secondary outcomes included wound status (open vs. closed) at postoperative day 14 (POD‐14), 1 month, 6 months, and 1 year, as well as patient and nurse acceptability regarding treatment comfort, difficulty with mobilization, and pain at the surgical site. These acceptability surveys were administered at 7 and 14 days following definitive closure.

### 2.5 Data Analysis

The effects of the FIS, the control, with and without ClimateCare in PUs undergoing operative closure were compared by determining the open versus closed status of the wound and complication rate between the two groups. Outcomes from nurse and subject acceptability surveys were analyzed and compared between both treatment arms. Categorical variables were summarized by frequencies and assessed for differences between groups using *χ* ^2^ analyses, Z‐scores, and odds ratios. Two‐sided tests were used and 0.05 was used to determine statistical significance. In addition, a power analysis was conducted to ensure sufficient statistical power to detect differences between the groups. The Cohen's *h* was calculated to assess the effect size for the difference in complication rates between the groups.

## 3 Results

A total of 38 patients completed the study, where 20 patients received the ClimateCare treatment and 18 patients did not. For both groups of patients, the mean BMI was similar at 25.8 ± 6.4 for the control and 25.7 ± 2.8 for the ClimateCare group. For the control group, the mean age of patients was 44.8 ± 11.8 years and 78% of patients were overweight or had obesity. Mean wound length was 7.0 ± 4.4 cm, mean wound width was 5.0 ± 3.3 cm, and mean wound depth was 2.4 ± 1.0 cm. For the ClimateCare group, the mean age of patients was 53.9 ± 14.0 years and 90% of patients were overweight or obese. Mean wound length was 4.7 ± 3.0 cm, mean wound width was 3.1 ± 1.9 cm, and mean wound depth was 2.8 ± 2.3 cm.

### 3.1 Complications

**Figure 1:**
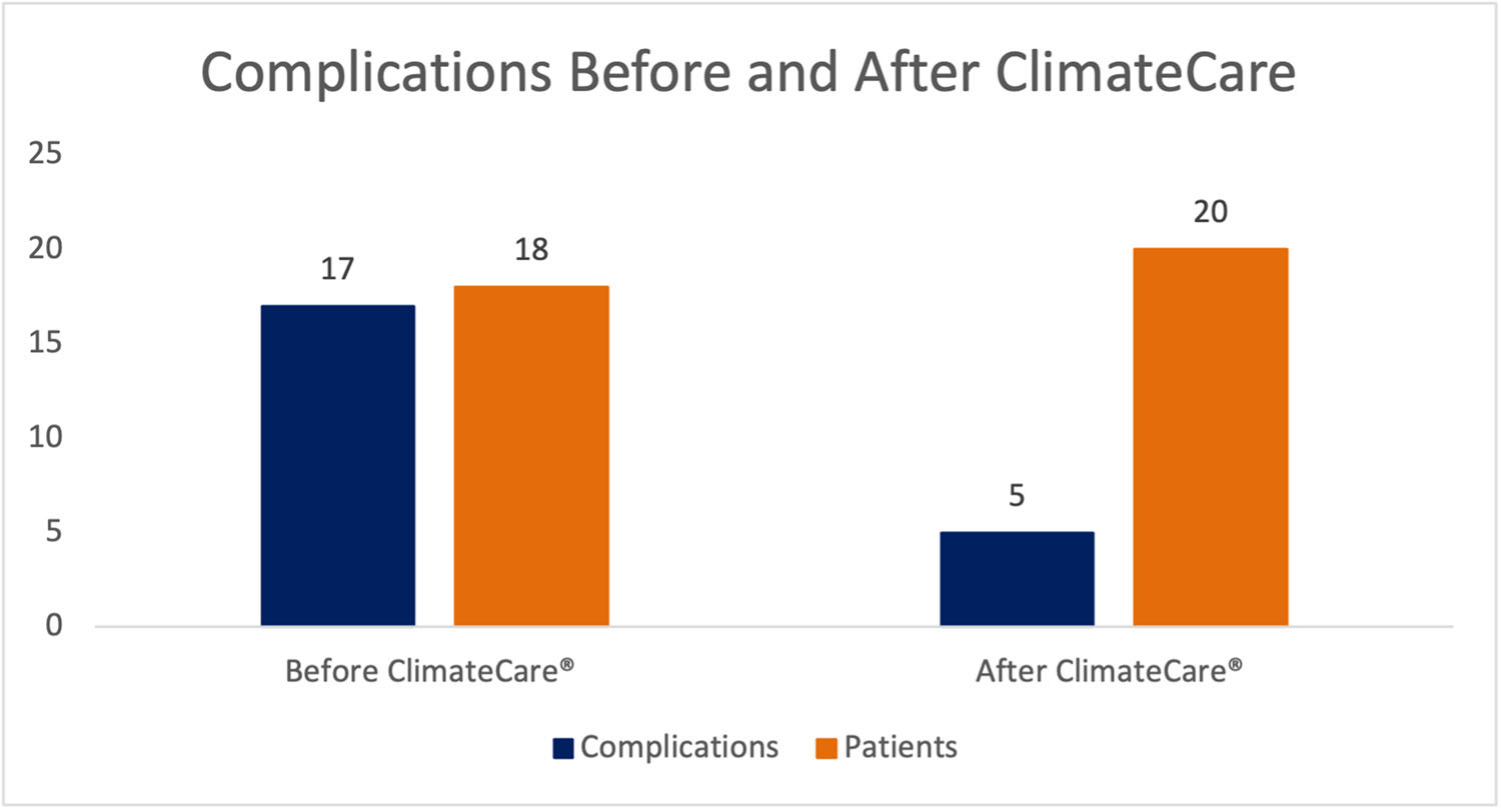
Complications before and after ClimateCare.

In the control group, 56% of patients had complications while 15% had complications in the ClimateCare group (*p* = 0.001) (Table 1, Figure 1). Complications for the control group included minor dehiscence (*n* = 7), maceration (*n* = 5), drainage (*n* = 2), major dehiscence (*n* = 1), congestion (*n* = 1), and moist area (*n* = 1), totalling 17 complications. Complications in the ClimateCare group included skin necrosis (*n* = 2), epidermolysis (*n* = 1), drainage (*n* = 1), and congestion (*n* = 1), totalling 5 complications. A *χ* ^2^ analysis revealed a significant difference in the occurrence of complications between the ClimateCare and control group (*p* = 0.03). The control group demonstrated a significantly higher occurrence of complications (56%) compared to the ClimateCare group (15%) (*z* = 2.63, *p* = 0.004). The odds of complications occurring in the control group are 7.08 times higher than the odds in the ClimateCare group (OR = 7.08, CI:) and a relative risk of 3.7. The absolute risk reduction from ClimateCare compared with the control is 40.6%, with a number needed to treat of 2.47, which implies that for every 2.47 patients given ClimateCare, one additional patient was likely to avoid a complication that they would have experienced from the control. The power of the study was calculated to be approximately 0.99, indicating that the study was adequately powered to detect the observed differences in complication rates between the two groups.

**Table 1:** Outcomes of ClimateCare intervention: complications and wound status.

Postoperative data: ClimateCare vs. control			
	ClimateCare	Control	*p* value
Complications at POD‐14, pts (%)	3 (15)	10 (56)	0.004
Type of Complication, *n* (%)			0.032
Moist Area	0 (0)	1 (5.9)	
Congestion	1 (20)	1 (5.9)	
Maceration	0 (0)	5 (29)	
Minor dehiscense	0 (0)	7 (41)	
Major dehiscense	0 (0)	1 (5.9)	
Epidermolysis	1 (20)	0 (0)	
Drainage	1 (20)	2 (12)	
Skin necrosis	2 (40)	0 (0)	
Number of complications, *n* (%)			
1 complication	1 (33)	4 (40)	
2 complications	2 (67)	5 (50)	
3 complications	0 (0)	1 (10)	
Wound status at POD‐14			
Open	0 (0)	4 (33)	0.006
Wound status at 1‐month			
Open	4 (25)	6 (50)	0.086
Wound status at 6‐month			
Open	2 (13)	2 (9.1)	0.391
Wound status at 1‐year			
Open	3 (19)	2 (20)	0.469

### 3.2 Wound Status

**Figure 2:**
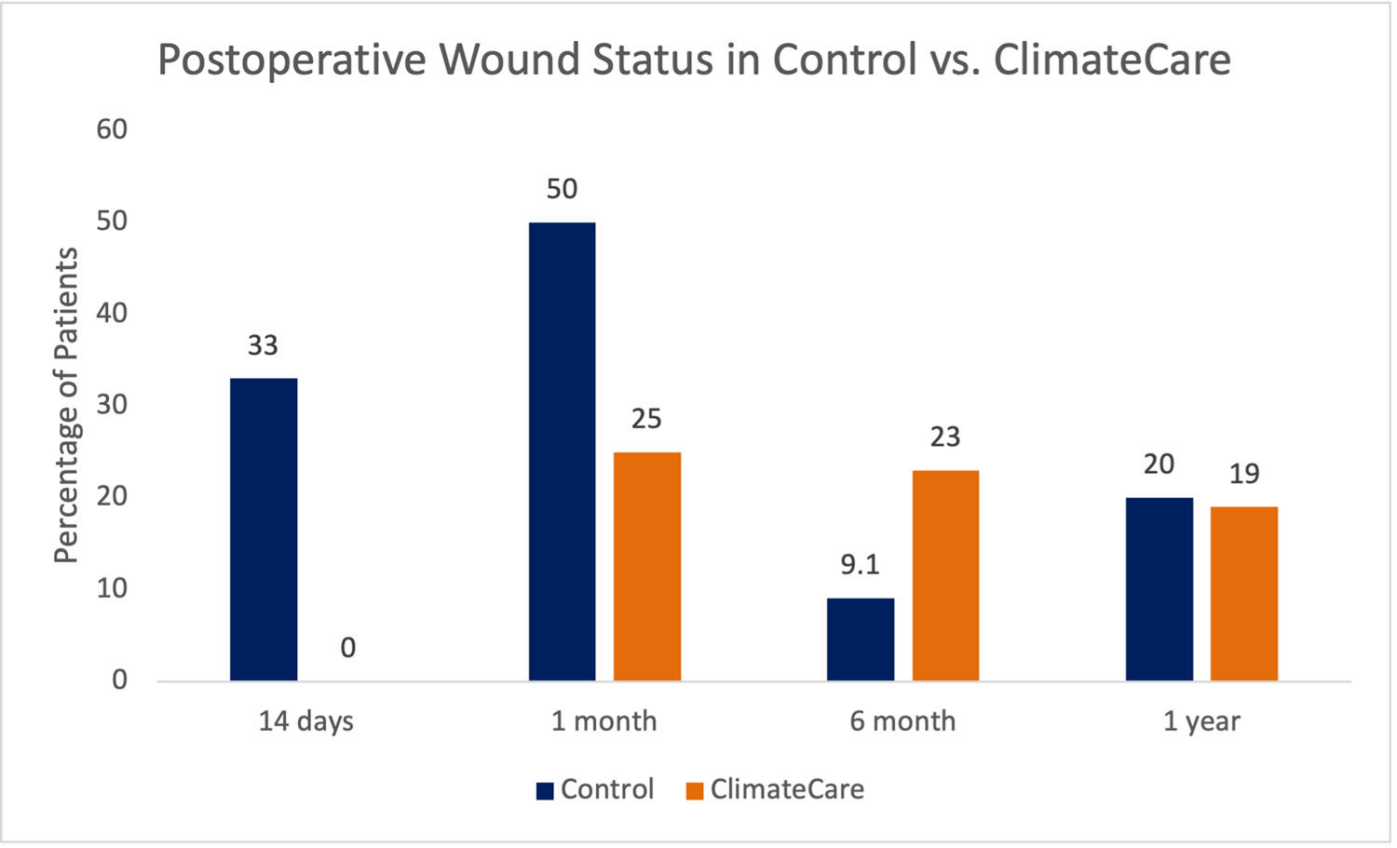
Wound status at POD14 before and after ClimateCare.

Thirty‐three percent of patients without ClimateCare had open wounds, while no patients who received ClimateCare treatment had open wounds at POD‐14, which was significantly different (*z* = 2.49, *p* = 0.01) (Figure 2). For patients without ClimateCare, 50% had open wounds at 1 month, 9.1% at 6 months, and 20% at 1 year. For patients with ClimateCare treatment, 25% had open wounds at 1 month, 13% at 6 months, and 19% at 1 year.

### 3.3 Patient and Nurse Reported Satisfaction

Patient acceptability regarding treatment comfort, difficulty with mobilization, and pain at the surgical site were not significantly different between ClimateCare and control groups.

## 4 Discussion

The standard of care to treat PUs has been generally well established; however, when it comes to postoperative wound care on supportive surfaces, the consensus is still inconclusive. Pressure ulcer care begins with prevention, primarily by frequent repositioning of patients and applying padding to decrease prolonged pressure over bony prominences. Additionally, keeping the skin clean and dry is crucial to prevent adverse outcomes. Repositioning, air‐fluidized mattresses, topical antiseptics, and intravenous antibiotics may be used to relieve existing pressure and manage existing infections. Aside from topical creams and various dressings, debridement may be necessary to remove dead or infected tissue [15]. The ClimateCare system is a relatively new and underutilized adjuvant therapy for managing pressure ulcer healing and preventing further ulcer formation. The system is a mattress cover that manages the microclimate of a patient's skin, with its therapeutic properties stemming from its high‐rate moisture vapor transfer system and ability to remove excess heat from tissue. Our findings provide evidence for the effectiveness of ClimateCare intervention in reducing the risk of complications and open wound status at POD‐14. The substantial relative risk decrease of 41% and low number needed to treat highlight the potential clinical significance of ClimateCare's impact on wound healing. We must consider the potential confounders and further explore the mechanisms underlying these results.

### 4.1 Climatecare and Wound Status

The ClimateCare group had no open wounds postoperatively whereas 33% of the control group experienced this adverse outcome. This difference exemplifies the benefit of microclimate control in pressure ulcer management risk reduction. Humidity, temperature, and airflow conditions cumulatively factor into the microclimate and thus impact skin deformation and susceptibility to wound formation [16, 17]. For instance, increased local humidity disturbs the stratum corneum, reducing stiffness and strength thereby decreasing tissue integrity [17, 18, 19, 20]. Notably, increased humidity and hydration of the stratum corneum is directly correlated with a higher coefficient of friction, meaning that skin experiences greater stresses from shear forces, such as when a patient repositions in a hospital bed [21, 22, 23, 24]. Additionally, the stratum corneum is sensitive to humidity changes, and increased hydration can allow for the transfer of irritants into the skin [25]. Therefore, the current recommendation is to keep skin dry to reduce the friction coefficient and resultant tissue stress [22, 26, 27].

Increased skin temperature is another modifiable factor linked to a suboptimal tissue microclimate via improved skin hydration and reduced cutaneous resistance [17, 28, 29]. Yusuf et al. found that increased skin temperature was associated with the development of pressure ulcers [30]. This was further supported by Yoshimura et al. who found that elevated skin temperature was an independent risk factor for the development of pressure ulcers that were acquired intraoperatively [31]. Such raised temperatures may also allow water to collect on the skin surface or permeate the stratum corneum [25]. ClimateCare reduces humidity and decreases temperature thus contributing to a more optimal microclimate that bolsters skin strength and helps maintain tissue integrity during wound healing [32]. In the present study, ClimateCare successfully facilitated healing and prevented the formation of open wounds by optimizing the humidity and temperature of the wound microclimate, establishing itself as a promising addition to patient care. Overall, the tissue microclimate can modulate the manifestation of wound complications and is a key factor in soft tissue deformation and resulting pressure ulcer formation.

### 4.2 Future Implications

Although the results from the present analysis are promising for the implementation of ClimateCare, many patient‐specific factors still need to be investigated in the context of pressure ulcer postoperative care. Specifically, patient age and location of pressure ulcer can influence the microclimate and healing process, necessitating the tailoring of therapeutics [25, 29, 33, 34, 35]. Given the similar patient acceptability between ClimateCare and standard of care protocol, ClimateCare is a feasible treatment measure which should continue to be evaluated for its effectiveness in pressure ulcer injury wound care, specifically in studies with a larger sample size and with an ability to stratify patients based on age, pressure ulcer location, and comorbidities. This will serve as a more rigorous assessment of ClimateCare, allowing for the adjustment of patient‐specific factors that can modulate wound healing processes. Overall, understanding how patient‐specific features interact with microclimate optimization in the postoperative setting of pressure ulcer care can assist with the formulation of personalized therapies with increased clinical utility.

### 4.3 Limitations

Although our study has provided evidence to support the effectiveness of ClimateCare in postoperative pressure ulcer management, it is not without limitations. The generalizability of the study's findings is limited due to the small sample size and the single‐center design. A larger, multi‐center study would be necessary to confirm these results and ensure their applicability to a broader population. In addition, the method of convenience sampling for treatment determination potentially introduces selection bias. Patient heterogeneity and variations in wound size may have also affected results. Although our study was able to capture complications within a year postoperatively, a longer follow‐up period could have better accounted for extended outcomes. Further evaluation of the effectiveness of ClimateCare should be done in larger and more diverse cohorts for extended periods of time.

## Author Contributions

**Stuti P. Garg:** conceptualization, data curation, investigation, methodology, validation, visualization, writing – original draft, writing – review and editing. **Anitesh Bajaj:** conceptualization, investigation, validation, visualization, writing – original draft, writing – review and editing. **Emmanuelle Hanna:** conceptualization, investigation, writing – original draft, writing – review and editing. **Geneviève L. Putnam:** conceptualization, investigation, validation, visualization, writing – original draft, writing – review and editing. **Iris Bai:** conceptualization, investigation, writing – original draft, writing – review and editing. **Diana Griffin:** conceptualization, investigation, writing – original draft, writing – review and editing. **Robert D. Galiano:** conceptualization, investigation, methodology, project administration, resources, supervision, writing – review and editing.

## Disclosure

All authors have read and approved the final version of the manuscript. Robert Galiano had full access to all of the data in this study and takes complete responsibility for the integrity of the data and the accuracy of the data analysis. No financial assistance was received in support of the study. Robert Galiano has the following disclosures: Peri and MTF Biologics. The funders had no role in the design of the study; in the collection, analyses, or interpretation of data; in the writing of the manuscript; or in the decision to publish the results. No other disclosures by any of the authors.

## Transparency Statement

The lead author Robert D. Galiano affirms that this manuscript is an honest, accurate, and transparent account of the study being reported; that no important aspects of the study have been omitted; and that any discrepancies from the study as planned (and, if relevant, registered) have been explained.

## Data Availability

The data that support the findings of this study are available from the corresponding author upon reasonable request.
